# Evaluation of myopia status and eye use behavior in school-age and preschool children

**DOI:** 10.1371/journal.pone.0322569

**Published:** 2025-06-11

**Authors:** Xiaolian Xie, Juan Ma, Qi Chen, Xiuna Li, Leina Jia, Juan Cao

**Affiliations:** 1 Department of Health and Health Education for Children and Adolescents, School of Public Health, Ningxia Medical University, Yinchuan, China; 2 Central Sterile Supply Department, Ningxia People’s Armed Police Corps Hospital, Yinchuan, China; 3 Key Laboratory of Environmental Factors and Chronic Disease Control, Ningxia Medical University, Yinchuan, China; University of Huddersfield, UNITED KINGDOM OF GREAT BRITAIN AND NORTHERN IRELAND

## Abstract

**Background:**

The aim of this research was to assess the incidence of myopia and associated behavioral risk factors among school-aged and preschool children in Ningxia.

**Methods:**

Our survey conducted a comprehensive cross-sectional study utilizing questionnaires to investigate the risk factors for myopia in children aged 3–10, both in preschool and school-age, based on parental reports of their children’s myopia status. The logistic regression model analysis was performed using myopia as dependent variables.

**Results:**

In Ningxia, the prevalence of myopia among preschool and school-age children stood at 4.9%. Specifically, the rate for school-age children (6.3%) was notably higher than that for preschool children (3.7%), with the difference being statistically significant (*P* < 0.05). The results of the logistic regression analysis indicated that senior grade, parental myopia, academic stress, playing electronic products, reading e-books for a long time, eye fatigue and less outdoor activities were risk factors for myopia, while not staying up late and reasonable reading distance (33–35 cm) were protective factors for myopia.

**Conclusions:**

The incidence of myopia among children aged 3–10 in Ningxia is 4.9%. Additionally, a child’s likelihood of developing myopia is influenced by whether their parents have myopia. Reading e-books and long-term exposure to electronic products are associated with myopia; Eye fatigue and academic stress are also important factors affecting myopia.

## Introduction

Myopia, also known as near-sightedness [1], has become a worldwide epidemic and stands as a primary cause of blindness and visual impairments [2]. The surge in myopia is expanding on a global scale, with projections indicating that 49.8% (4.76 billion) of the world’s population will be affected by myopia in 2050 [3]. Myopia also poses a significant economic burden. The Ma Y [4] study also showed that the total economic burden of myopia was estimated as 26.3 billion US$. The rising incidence of myopia during early childhood has elevated the likelihood of progressing to high myopia and associated vision-impairing ocular conditions in adulthood [5]. Therefore, it is of importance to postpone myopia onset and slow myopia progression in school-age and preschool children. Studies have reported that the prevalence of myopia among preschool children (aged below 7 years) exceeds 5% [6]. The significant increase in myopia rates, which is occurring despite a relatively stable genetic backdrop, highlights the profound impact of environmental and lifestyle factors on this condition [7].

Currently, there is a scarcity of large-scale studies examining the factors influencing myopia in both school-age and preschool children. To address this gap, the present study employed a questionnaire survey to elucidate the risk factors associated with myopia in these age groups. Such insights will aid in the development of more effective and tailored interventions to curb the escalating rates of childhood myopia globally.

## Materials and methods

### Participants

From October to December 2023, a stratified cluster random sampling method was used to randomly select 400 kindergartens and 140 primary schools across both urban and rural areas in 5 cities of Ningxia (Yinchuan, Wuzhong, Shizuishan, Guyuan, and Zhongwei). To ensure representative coverage, the sampling framework included districts and counties within each city, encompassing diverse socioeconomic and geographic settings. All students in kindergartens and all students in grades 1–3 of primary schools were selected as subjects. Primary caregivers of the children involved in the study were requested to fill out a structured questionnaire, encompassing details about the child’s age, grade, sleep patterns, physical activity levels, screen time, and their parents’ refractive status. Questionnaires were excluded if: (1) the child suffered from a visual impairment other than myopia (n = 2,107); (2) there was incomplete information regarding ocular behaviors (n = 4,252). A total of 6,359 children (6.7% of the initial sample) were excluded, resulting in a final analytical sample of 88,534 participants. The excluded participants did not differ significantly from the included cohort in terms of age or gender distribution (*p* > 0.05). The research was granted approval by the Ethics Committee of Ningxia Medical University (Approval No. 2022-N057), and written informed consent was secured from the guardians of all participating children.

### Determination of myopia status

The following questions pertaining to the children’s ocular health were included in the survey [8]: (1) Has your child ever been diagnosed with visual impairment by an ophthalmologist? (0 = ‘no’, 1 = ‘yes’, 2 = ‘unclear’); (2) If ‘yes’, participants were further queried about the specific diagnosis, including astigmatism, myopia, hyperopia, strabismus, amblyopia, or other common visual issues. For this study, the analysis was confined to a total of 88,534 children who either had no visual impairment or were reported to have been diagnosed solely with myopia.

### Statistical analyses

For the bivariate analysis, categorical variables were assessed using the Pearson chi-square test. To identify the factors influencing myopia in school-age and preschool children, variables that demonstrated statistical significance (P < 0.05) in the initial analysis were incorporated into a logistic regression model. This model was utilized to estimate the odds ratios (*ORs*) and corresponding 95% confidence intervals (*CIs*) or p values. Statistical significance was set at a two-sided p value of less than 0.05. All statistical analyses were conducted using R software, version 4.3.2.

## Results

### Prevalence of myopia among school-age and preschool children

A comprehensive total of 88,534 children participated in this study, comprising 48,829 preschool children aged 3–6 years (representing 55.2% of the sample) and 39,705 school-age children aged 7–10 years (accounting for 44.8% of the sample). The overall prevalence of myopia was found to be 4.9% (4,322 out of 88,534 children), with a prevalence of 3.7% (1,824 out of 48,829) among preschool children and 6.3% (2,498 out of 39,705) among school-age children. This difference was statistically significant (*χ*^2^ = 308.08, p < 0.001). Further details are presented in Table 1.

**Table 1:** Prevalence of myopia in school-age (7–10 years) and preschool (3–6 years) children.

Classification	Age (years)	No. of Children	Percentage (%)	No. with Myopia	Myopia Prevalence (%)
**Preschool children**	3	5923	6.7	242	4.1
	4	12991	14.7	488	3.8
	5	13030	14.7	494	3.8
	6	16885	19.1	600	3.6
**School-age children**	7	18140	20.5	830	4.6
	8	14835	16.8	1090	7.3
	9	6534	7.4	561	8.6
	10	196	0.2	17	8.7
**Total**		88534	100	4322	4.9

### Correlations between the described attributes of children and the occurrence of myopia

In this study, 21 myopia related variables were included for univariate analysis, and the results showed that except for dietary factor, the other 20 factors were related to myopia (all P values <0.05). Additional information is provided in Table 2.

**Table 2:** Bivariate correlations between grade, ocular behaviors, and myopia occurrence in children (N = 88,534).

Characteristics		Myopia (n/%)		*χ* ^2^	*P* value
		No	Yes		
**Grade**	Kindergarten	34679 (96.2)	1383 (3.8)	657.67	<0.001
	First grade	17859 (96.5)	656 (3.5)		
	Second grade	15936 (95.2)	810 (4.8)		
	Third grade	15738 (91.4)	1473 (8.6)		
**Parental myopic status**	None	44314 (98.3)	779 (1.7)	2018.44	<0.001
	One	28930 (92.3)	2415 (7.7)		
	Both	10968 (90.7)	1128 (9.3)		
**Academic stress**	No pressure	49136 (97.3)	1377 (2.7)	1259.72	<0.001
	Moderate pressure	31853 (92.6)	2553 (7.4)		
	High pressure	3223 (89.2)	392 (10.8)		
**Have the habit of often staying up late?**	Yes	5961 (86.3)	943 (13.7)	1242.27	<0.001
	No	78251 (95.9)	3379 (4.1)		
**Daily exposure to electronic products(hours)**	0–1	64435 (96.2)	2527 (3.8)	1872.01	<0.001
	1 ~ 3	16420 (94.1)	1031 (5.9)		
	≥3	3357 (81.5)	764 (18.5)		
**Tools for Reading**	Electronic book	7447 (88.6)	959 (11.4)	852.07	<0.001
	Paper book	76765 (95.8)	3363 (4.2)		
**Do you often feel eye fatigue?**	Often	2487 (78.6)	678 (21.4)	3647.30	<0.001
	Sometimes	29306 (91.8)	2624 (8.2)		
	Seldom	52419 (98.1)	1020 (1.9)		
**Perform eye exercises**	No	32524 (94.7)	1806 (5.3)	17.34	<0.001
	Yes	51688 (95.4)	2516 (4.6)		
**Participate in physical exercise and increase outdoor activities**	No	38113 (94.9)	2030 (5.1)	4.85	0.028
	Yes	46099 (95.3)	2292 (4.7)		
**Whether you regularly consume foods rich in vitamin A (e.g., carrots, spinach, animal liver)**	No	39227 (95.1)	2034 (4.9)	0.38	0.537
	Yes	44985 (95.2)	2288 (4.8)		
**Pay attention to the sitting position and keep the distance between the eyes and the book at about 33–35 cm**	No	26925 (93.7)	1805 (6.3)	179.76	<0.001
	Yes	57287 (95.8)	2517 (4.2)		
**In school, I get out of the classroom and exposure to natural light for more than 1 hour every day**	No	21853 (93.1)	1614 (6.9)	273.96	<0.001
	Yes	62359 (95.8)	2708 (4.2)		
**Outside of school, I was exposed to natural light for more than 1 hour a day, such as walking or playing outdoors**	No	20212 (93.2)	1482 (6.8)	235.23	<0.001
	Yes	64000 (95.8)	2840 (4.2)		
**During the recess, I will take the initiative to go out of the classroom**	No	21001 (93.3)	1510 (6.7)	216.78	<0.001
	Yes	63211 (95.7)	2812 (4.3)		
**When using the computer, my eyes are more than 50 cm away from the computer display**	No	16242 (92.6)	1306 (7.4)	309.06	<0.001
	Yes	67970 (95.8)	3016 (4.2)		
**I have at least one hobby of outdoor sports or exercise**	No	19329 (92.8)	1495 (7.2)	309.52	<0.001
	Yes	64883 (95.8)	2827 (4.2)		
**After 40 minutes of continuous reading and writing, I relax my eyes in one or more ways (looking away or moving lightly or outdoors or doing eye exercises)**	No	24819 (93.0)	1856 (7.0)	354.38	<0.001
	Yes	59393 (96.0)	2466 (4.0)		
**In winter and summer vacation, I can insist on physical exercise**	No	28310 (93.6)	1930 (6.4)	222.70	<0.001
	Yes	55902 (95.9)	2392 (4.1)		
**I get 8 hours of sleep a day**	No	7923 (90.7)	809 (9.3)	400.79	<0.001
	Yes	76289 (95.6)	3513 (4.4)		
**The average time I wake up on weekends is usually no more than an hour compared to school days**	No	16214 (92.9)	1230 (7.1)	220.18	<0.001
	Yes	67998 (95.7)	3092 (4.3)		
**When I read and write, my eyes are more than a foot (33 cm) away from the book, my chest is about a punch from the edge of the table, and my fingers are about an inch (3.3 cm) away from the tip of the pen**	No	25334 (92.9)	1932 (7.1)	412.18	<0.001
	Yes	58878 (96.1)	2390 (3.9)		

### Multivariate analysis of influencing factors of myopia in school-age and preschool children

In order to further analyze the risk factors of myopia in school-age and preschool children, we included 20 variables with statistical significance in univariate analysis (Table 2) into logistic regression analysis. The results showed that 9 variables were ultimately related to myopia. Among them, senior grade, parents ‘ myopia, academic pressure, playing electronic products, reading electronic books for a long time, eye fatigue and less outdoor activities are risk factors for myopia, while not staying up late and reasonable reading distance (33 ~ 35 cm) are protective factors.

Compared with parents with normal vision, the odds ratio (OR) of giving birth to a myopic child was 4.70 (4.31 to 5.11) when one parent had myopia and 6.10 (5.52 to 6.74) when two parents had myopia. With the increase of eye fatigue frequency, the risk of myopia increased (OR value increased from 2.86 to 5.25). And the adjusted OR (95%CI) increased from 1.16 (1.07,1.26) to 2.81 (2.51,3.15) as the screen use rose from 0–1 hour to 3 hours. Compared with reading paper books, reading e-books increased the risk of myopia by 1.71 (1.55 ~ 1.88). In addition, staying up late is also associated with myopia, showing that children who do not stay up late have a lower risk of myopia(OR=0.75, 0.69 ~ 0.83) (Table 3).

**Table 3:** Multivariable logistic regression analysis of factors associated with myopia in school-age and preschool children.

Characteristics	B	SE	Wald	*P* Value	OR Value	95%CI	
**Lower**	**Upper**
**Intercept**	−2.964	0.103	825.315	<0.001	0.052		
**Grade**			500.885	<0.001			
** Kindergarten**	Reference						
** First grade**	−0.018	0.054	0.111	0.739	0.982	0.884	1.091
** Second grade**	0.27	0.053	26.121	<0.001	1.31	1.181	1.453
** Third grade**	0.919	0.049	355.871	<0.001	2.508	2.279	2.759
**Parental myopic status**			1549.861	<0.001			
** None**	Reference						
** One**	1.546	0.043	1270.255	<0.001	4.695	4.312	5.111
** Both**	1.808	0.051	1274.581	<0.001	6.10	5.523	6.736
**Academic stress**			155.822	<0.001			
** No pressure**	Reference						
** Moderate pressure**	0.496	0.04	155.282	<0.001	1.643	1.519	1.776
** High pressure**	0.42	0.071	35.201	<0.001	1.522	1.325	1.749
**Have the habit of often staying up late?**							
** Yes**	Reference						
** No**	−0.283	0.049	33.935	<0.001	0.754	0.686	0.829
**Daily exposure to electronic products(hours)**			322.481	<0.001			
** 0 ~ 1**	Reference						
** 1 ~ 3**	0.149	0.042	12.724	<0.001	1.16	1.069	1.259
** ≥ 3**	1.034	0.058	316.016	<0.001	2.812	2.509	3.152
**Tools for Reading**							
** Paper book**	Reference						
** Electronic book**	0.536	0.049	117.865	<0.001	1.709	1.551	1.882
**Do you often feel eye fatigue?**			871.748	<0.001			
** Seldom**	Reference						
** Sometimes**	1.05	0.041	657.306	<0.001	2.857	2.637	3.096
** Often**	1.658	0.064	665.548	<0.001	5.248	4.627	5.952
**Pay attention to the sitting position and keep the distance between the eyes and the book at about 33–35 cm**							
** No**	Reference						
** Yes**	−0.155	0.036	18.546	<0.001	0.857	0.799	0.919
**In school, I get out of the classroom and exposure to natural light for more than 1 hour every day**							
** Yes**	Reference						
** No**	0.101	0.045	5.111	0.024	1.106	1.014	1.207

B (unstandardized regression coefficient), SE (standard error), OR (odds ratio), 95% CI (confidence interval).

## Discussion

The current study revealed a myopia prevalence of 4.9%, with 3.7% among preschool children aged 3–6 years and 6.3% among school-age children aged 7–10 years. In contrast, a survey conducted by Yang GY [9] on 27,070 preschool children aged 3–7 years reported a myopia prevalence of 2.24%. Furthermore, a Japanese study in 2021 indicated a myopia rate of 2.9% among preschool children [10]. A meta-analysis [11] revealed that the prevalence of myopia in children aged 5–11 years in Africa was 3.4% (95% CI: 2.5–4.4%). Additionally, Li SM’s research [12] demonstrated that between 2012 and 2017, the prevalence of myopia increased from 6.6% (95% CI: 5.6–7.5%) in first-grade students to 25.2% (23.6–26.8%) in third-grade students. Consequently, the prevalence of myopia among children varies across different countries and regions, yet it remains consistently high, warranting our attention and concern.

Our study underscored the significance of parental myopia in determining the prevalence of myopia among students. Among the 4,322 individuals with myopia, 3,543 (82.0%) reported a parental history of myopia. Within this subgroup, 31.8%(1128/3543) had both parents affected by myopia, while 68.2% (2415/3543) had only one affected parent. Children with parental myopia exhibited a heightened risk of developing myopia. In comparison to children whose parents had normal vision, the risk of myopia was elevated by 4.7 times and 6.1 times in children with one and both parents being myopic, respectively. This is consistent with the results of related literature [13,14]. The possible reason is that several gene loci are related to axial elongation, but the complex interaction between individual genes, behavioral habits and risk factors for myopia progression in children needs further study [14].

Our study found that reading e-books and long-term exposure to electronic products were associated with myopia. Compared with children who read paper books, e-books increased the risk of myopia by 1.71 times. At the same time, when the use time of children ‘s electronic products increased to more than 1 hour and more than 3 hours, the risk of myopia increased by 1.16 times and 2.81 times, respectively. Similarly, a previous meta-analysis [15] found that for every additional hour of myopia work per week, the probability of myopia increased by 2%. These causes of myopia are related to near-vision work. The small screen and font of electronic products lead to a closer viewing distance. Considering that electronic devices work longer and closer than other forms of near vision, they may also produce similar myopia. Compared with paper books, it puts forward higher requirements for eye adjustment and convergence, resulting in excessive adjustment of convergence and axial extension effect of peripheral defocus [16]. In addition, the utilization of electronic devices diminishes the duration of outdoor activities and decreases the concentration of retinal dopamine due to reduced exposure to light, ultimately prompting an increase in axial eye growth and consequently leading to myopia [17,18].

Our study findings of no significant association between diet and incident myopia, which is consistent with Li M ‘s [19] study. The relationship between diet and myopia needs further study.

Eye fatigue is an important factor affecting myopia. Only 3.6% (3165/ 88534) of children in our study often feel eye fatigue, but the prevalence of myopia is as high as 21.4% (378/ 3165). Children who reported frequent eyestrain had a 5.25-fold increased risk of myopia compared to those who had less frequent eyestrain. Research has indicated that prolonged use of electronic devices leads to eye fatigue. The eye’s focusing and ocular movements necessary for clearer visibility of digital screens impose additional strain on the delicate equilibrium between accommodation and convergence mechanisms. Consequently, individuals with uncorrected or inadequately corrected refractive errors become even more vulnerable to this strain [8]. In addition, myopia, in turn, can cause eye fatigue [20]. However, some literature suggests that the symptoms of digital eye fatigue are transient and do not threaten vision [21]. At present, there is no clear evidence to prove the causal relationship between eye fatigue and myopia, which is worthy of attention in future research.

Academic stress is positively correlated with myopia. The greater the stress, the higher the prevalence of myopia. Some literature [22] has pointed out that myopia is a risk factor for stress. Students with myopia complained more about anxiety and stress. However, there is no relevant literature to report the relationship between academic stress and myopia, which requires further study.

This study carries certain limitations. Specifically, data regarding the factors influencing myopia were gathered through self-administered questionnaires completed by parents, potentially introducing recall bias. Nevertheless, the results of this study align with previous findings utilizing similar questionnaires and are consistent with the conclusions drawn from prior research. While our study incorporated both urban and rural populations within Ningxia, future research could further explore regional disparities by directly comparing myopia prevalence between urban and rural settings, accounting for potential confounders such as educational intensity and outdoor activity time.

## Conclusions

The prevalence of myopia among children aged 3–10 years in Ningxia reached a high level of 4.9%. Parental myopia has a significant impact on the incidence of myopia in children. In comparison to children whose parents have normal visual acuity, the risk of myopia increases by 4.7 times and 6.1 times in children with one and both parents being myopic, respectively; Reading e-books and long-term exposure to electronic products are associated with myopia; Compared with children who read paper books, the risk of myopia in e-books increased by 1.71 times; Eye fatigue and academic stress are also important factors affecting myopia.

## Supporting information

S1 File Minimal data set. (XLSX)
